# Neural Circuits Underlying Behavioral Flexibility: Insights From *Drosophila*

**DOI:** 10.3389/fnbeh.2021.821680

**Published:** 2022-01-06

**Authors:** Anita V. Devineni, Kristin M. Scaplen

**Affiliations:** ^1^Department of Biology, Emory University, Atlanta, GA, United States; ^2^Zuckerman Mind Brain Institute, Columbia University, New York, NY, United States; ^3^Department of Psychology, Bryant University, Smithfield, RI, United States; ^4^Center for Health and Behavioral Studies, Bryant University, Smithfield, RI, United States; ^5^Department of Neuroscience, Brown University, Providence, RI, United States

**Keywords:** *Drosophila*, environmental context, behavioral flexibility, neural circuits, internal state, behavioral state, learning, memory

## Abstract

Behavioral flexibility is critical to survival. Animals must adapt their behavioral responses based on changes in the environmental context, internal state, or experience. Studies in *Drosophila melanogaster* have provided insight into the neural circuit mechanisms underlying behavioral flexibility. Here we discuss how *Drosophila* behavior is modulated by internal and behavioral state, environmental context, and learning. We describe general principles of neural circuit organization and modulation that underlie behavioral flexibility, principles that are likely to extend to other species.

## Introduction

Across the animal kingdom, an animal’s ability to modulate behavior in response to changing internal and external conditions is critical for survival. For example, animals are often faced with the challenge of finding food or water while simultaneously avoiding dangerous situations. If a predator’s scent is detected, an animal might choose to hide. However, if they are hungry enough, they may prioritize finding food over staying hidden. Even a fruit fly is not always constrained by instinct but exhibits remarkable behavioral flexibility. For instance, carbon dioxide is released by fermenting fruit, a preferred food source for *Drosophila*, but may also represent a distress signal from other flies (Suh et al., 2004). Thus, when a fly smells carbon dioxide, it must decide: does it choose to seek a potential food source or avoid potential danger?

Behavioral flexibility can be defined as an animal’s ability to adapt its behavioral responses to changing environmental contingencies or internal state (Kolb, 1990; Ragozzino et al., 1999; Floresco et al., 2009; Lea et al., 2020). *Drosophila* *melanogaster* is a powerful model organism for investigating the neural circuits underlying behavioral flexibility. Not only do these insects exhibit remarkable flexibility in their behavior, but the repertoire of advanced genetic tools available to study neural circuits is simply unprecedented. Genetic approaches enable us to target individual neurons or cell types in order to manipulate or record their neural activity. In addition, the recently published synaptic connectome of the fly brain (Scheffer et al., 2020) has greatly facilitated neural circuit identification and analysis. Using these tools, work in *Drosophila* has revealed the mechanisms underlying many different examples of behavioral flexibility, from learning and memory to state-dependent modulation.

**Figure 1:**
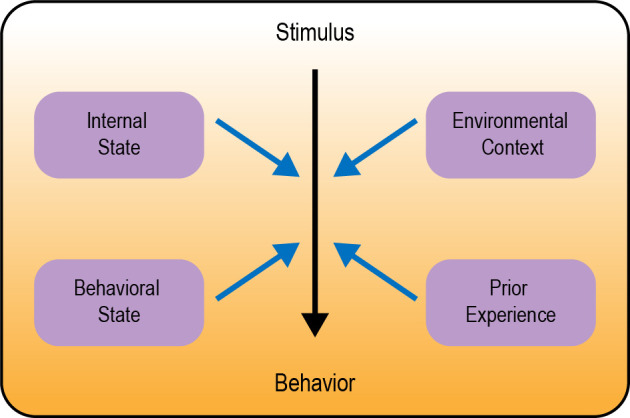
Overview of different types of behavioral modulation. Neural circuits in the brain transform a sensory stimulus into a behavioral response (represented by the color gradient). Black arrow depicts the core circuit underlying behavior; blue arrows depict modulation of this circuit.

In this review we describe several examples of behavioral flexibility in *Drosophila*, focusing on four major categories: (1) modulation by internal state; (2) modulation by behavioral state; (3) modulation by environmental context; and (4) behavioral flexibility in learning and memory (Figure 1). We focus specifically on studies of adult *Drosophila*. There are a number of articles describing behavioral flexibility in *Drosophila* larvae that are not discussed here (e.g., Schroll et al., 2006; Schleyer et al., 2011, 2020; Allen et al., 2017; Mancini et al., 2019; Eschbach et al., 2020; Miroschnikow et al., 2020; Slankster et al., 2020; Gowda et al., 2021; Hernandez-Nunez et al., 2021; Vogt et al., 2021). Our goal is to highlight generalizable neural circuitry frameworks for how sensory cues, state, and experience are integrated to guide the flexible selection of appropriate behavior.

## Modulation by Internal State

### Modulation by Hunger

The internal state of an animal can profoundly affect behavior. One such state is hunger, which is induced by energy deprivation. Hunger modulates a large repertoire of behaviors in order to promote food-seeking, food consumption, and, in some cases, to conserve energy. The impact of hunger state includes modulation of sensory processing as well as downstream pathways regulating locomotor and choice behaviors, as described below.

**Figure 2:**
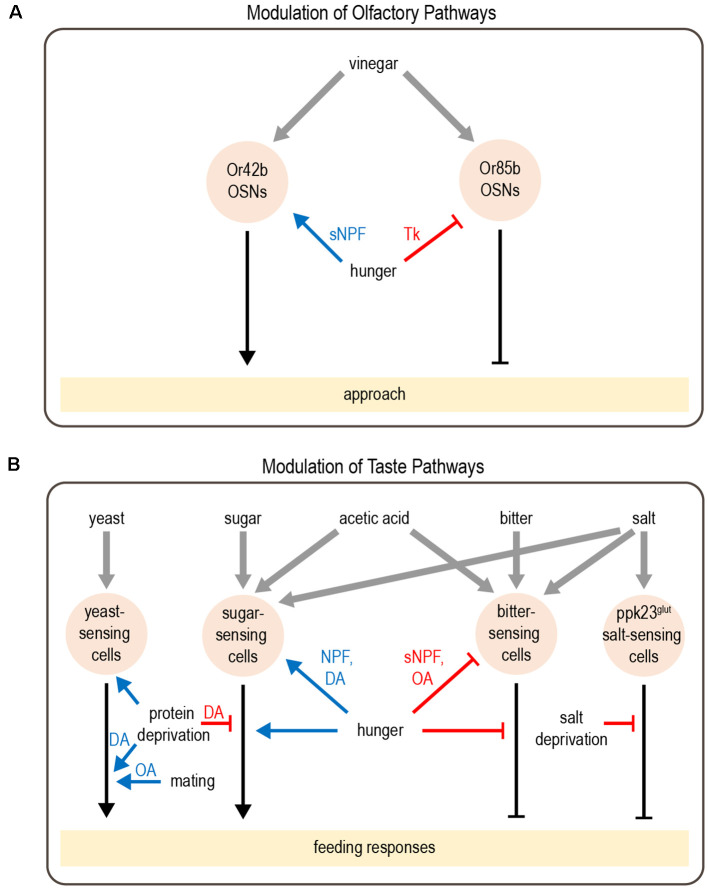
Modulation of olfactory and taste pathways by internal states. **(A)** Parallel olfactory pathways are modulated by hunger at the level of sensory neuron output. **(B)** A variety of taste pathways are modulated by states such as hunger, protein or salt deprivation, or mating. This modulation can act at the level of sensory neurons or downstream. Most downstream neuronal targets of modulation have not yet been identified. Abbreviations not defined in text: tachykinin (Tk); octopamine (OA); dopamine (DA). Gray arrows depict sensory input; black arrows depict neural circuits that generate behavior; blue arrows depict excitatory modulation whereas red arrows depict inhibitory modulation.

Several studies have revealed how hunger modulates sensory perception in flies. This often involves the modulation of parallel pathways that mediate opposing responses. Flies rely on smell to identify and navigate toward potential food sources. Olfactory detection is mediated by a large number of olfactory sensory neurons (OSNs) in the antenna and maxillary palp, which project to the antennal lobe of the brain (Montell, 2021). Different classes of OSNs express different olfactory receptors, which determine the set of odorants to which the neuron responds (Vosshall et al., 2000; Hallem et al., 2004; Fishilevich and Vosshall, 2005; Hallem and Carlson, 2006; Su et al., 2009). Olfactory attraction to vinegar, a natural food source, reflects a balance between two competing pathways: an attractive pathway mediated by Or42b-expressing OSNs and an aversive pathway mediated by Or85b-expressing OSNs, engaged at high vinegar concentrations (Semmelhack and Wang, 2009; Root et al., 2011; Ko et al., 2015). Hunger promotes attraction to vinegar by both enhancing the attractive olfactory pathway and suppressing the aversive olfactory pathway (Figure 2A). These parallel actions utilize distinct neuromodulatory and circuit mechanisms. The attractive OSNs release short neuropeptide F (sNPF), which acts in an autocrine manner to enhance their presynaptic activity (Root et al., 2011). In contrast, the activity of aversive OSNs is suppressed by tachykinin released from local interneurons (Ko et al., 2015). Both sNPF and tachykinin signaling in OSNs are regulated by insulin signaling, which represents a global satiety signal (Root et al., 2011; Ko et al., 2015).

Hunger-dependent modulation of food-seeking behaviors also relies on the integration of hunger and satiety signals in downstream processing centers. One such site is the mushroom body (MB), a high-level integration center essential for learning and memory (Heisenberg et al., 1985; Davis, 1993; de Belle and Heisenberg, 1994; Heisenberg, 1998, 2003; Zars, 2000; Pascual and Preat, 2001). Tsao et al. (2018) showed that a diverse group of hunger and satiety signals, including insulin, serotonin, and sNPF, regulate the activity of dopaminergic neurons (DANs) that innervate the MB. The activity of specific MB output neurons (MBONs) are modulated by hunger, likely *via* inputs from DANs, and these MBONs promote food-seeking when flies are hungry (Tsao et al., 2018). Once flies successfully find food, they should stop food-seeking in order to prioritize other behaviors. This behavioral switch is also mediated by the MB: in the presence of food, the persistence of food-seeking is inhibited by octopaminergic neurons innervating the MB (Sayin et al., 2019).

Similar to hunger-dependent modulation of olfaction, parallel modulation of opposing pathways also underlies hunger-dependent changes in taste sensitivity. Flies rely on taste in making the final decision about whether to consume food. Most studies of taste have focused on sweet and bitter tastes, which are detected by separate populations of neurons that promote or inhibit feeding, respectively (Montell, 2021). Hunger enhances taste sensitivity to sugar (Inagaki et al., 2012; Marella et al., 2012), which promotes energy consumption during this time of energy deficit. In parallel, hunger decreases bitter sensitivity (Inagaki et al., 2014), which increases a fly’s willingness to consume food containing bitter-tasting contaminants. Like olfactory modulation, taste sensitivity is modulated at the level of sensory neurons and different channels are modulated by distinct mechanisms (Figure 2B). Dopamine release during hunger enhances the presynaptic activity of sugar-sensing neurons, which relies on upstream neuropeptide F (NPF) signaling (Inagaki et al., 2012, 2014). In contrast, hunger decreases octopamine signaling to suppress the activity of bitter-sensing neurons. This requires sNPF but not NPF signaling (Inagaki et al., 2014; LeDue et al., 2016).

In addition to modulating the strength of taste attraction or aversion, hunger can also elicit a behavioral switch in the taste response. Acetic acid is a natural food source that provides calories, but it can also be toxic (Parsons, 1980; Hoffman and Parsons, 1984). Fed flies show taste aversion to acetic acid, whereas hungry flies show a strong appetitive response (Devineni et al., 2019). Acetic acid activates both the sugar- and bitter-sensing pathways, which respectively promote feeding attraction or aversion. The balance between these pathways determines the behavioral response. Hunger shifts this balance by enhancing the sugar-sensing pathway as well as suppressing the bitter pathway, resulting in a behavioral switch from aversion to attraction (Figure 2B). Although this modulation is consistent with the changes in sugar and bitter sensitivity described above, in this study the activity of taste sensory neurons showed very little modulation by hunger. Modulation of downstream taste pathways is therefore likely to be involved.

A theme that emerges is that, during hunger, distinct mechanisms function in parallel to modulate attractive and aversive food-sensing pathways. This affords greater control and flexibility in modulating behavior. For example, having distinct mechanisms for modulating sugar and bitter sensitivity allows these pathways to be modulated on different timescales (Inagaki et al., 2014). Mild starvation over short timescales (within 6 h) enhances sugar sensitivity, which represents a low-risk behavioral change. In contrast, prolonged starvation (at least 24 h) is required to decrease bitter sensitivity, which is a high-risk change given that bitter compounds may be toxic. In olfaction, the aversive pathway for sensing vinegar is primarily engaged at high vinegar concentrations (Semmelhack and Wang, 2009; Ko et al., 2015), which are more likely to be toxic. Parallel modulation of the attractive and aversive pathways thus allows for differential modulation of vinegar attraction depending on its concentration.

In addition to modulating food-seeking behavior, another important survival strategy during starvation is to reduce one’s metabolic rate in order to save energy. In ectothermic organisms such as *Drosophila*, metabolic rate depends on environmental temperature and can be reduced by moving to cooler temperatures. Indeed, starvation lowers a fly’s preferred temperature by 2–3°C (Umezaki et al., 2018). Hunger elicits this behavioral change by modulating a specific set of thermosensory neurons, the anterior cells (AC). In starved flies, the ACs are activated by lower temperatures, which may lower the set point for the fly’s preferred temperature (Umezaki et al., 2018). Unlike the changes in olfactory and taste sensitivity described above, the effect of hunger on ACs represents a tuning change rather than a gain change: the sensory neurons’ preferred stimulus changes but their peak response amplitude remains constant. While gain changes can up- or downregulate specific behavioral responses, tuning changes provide a clearer mechanism to alter an animal’s preference as it chooses between different stimuli of the same type.

Beyond modulation of sensory perception, hunger acts on central circuits to alter locomotor activity. Starved flies show increased locomotion (Lee and Park, 2004; Isabel et al., 2005), which may represent an enhanced exploratory drive and increase the likelihood of finding food. This change relies on bidirectional modulation by opposing metabolic signals, insulin (a satiety signal), and adipokinetic hormone (AKH, a hunger signal considered the analog of mammalian glucagon; Yu et al., 2016). AKH acts on a set of octopaminergic cells to promote hyperactivity during starvation. Conversely, insulin suppresses locomotor activity during satiety. AKH and insulin regulate locomotor activity by acting on the same set of cells, and this bidirectional regulation by hunger and satiety signals may ensure more robust control over behavior (Yu et al., 2016). Interestingly, the octopaminergic neurons that promote starvation-induced hyperactivity are not required for increased food consumption in starved flies, revealing that parallel mechanisms control distinct hunger-regulated behaviors (Yu et al., 2016).

Parallel regulation of different pathways thus emerges as a general principle for hunger modulation of behavior. As discussed above, the modulation of parallel pathways may allow for more flexibility. For example, behavior can be regulated on different timescales to promote different behavioral changes depending on whether an animal is facing mild or severe starvation. Another emerging principle is the role of slow-acting neurotransmitters and neuropeptides in hunger modulation. The release of neuromodulators during hunger or satiety states allows for state-dependent modulation of specific neural circuits that express the appropriate receptors. The strength of the modulation can therefore be tuned globally by altering the amount of neuromodulator that is released or locally by altering receptor expression levels.

### Modulation by Other States Reflecting Changing Nutrient Demands

Although starvation has received the most attention, behavior is also modulated by other states reflecting changing nutrient demands. One example is the modulation of salt consumption. Similar to mammals, flies show taste attraction to low concentrations of salt, an essential nutrient, but avoid it at high concentrations. Depriving flies of salt reduces their aversion to high salt concentrations (Jaeger et al., 2018). Like acetic acid, salt activates multiple classes of taste sensory cells, including sugar- and bitter-sensing neurons as well as a population of Ppk23-expressing glutamatergic (Ppk23^glut^) neurons that seem to be specific for salt-sensing. Salt deprivation specifically modulates the Ppk23^glut^ taste pathway, ensuring that salt preference is modulated without affecting responses to sugar or bitter (Figure 2B). Activating Ppk23^glut^ cells elicits lower salt aversion in salt-deprived flies than controls, demonstrating that state-dependent modulation occurs downstream of sensory cells (Jaeger et al., 2018).

Animals must also balance multiple nutritional needs, such as the need for carbohydrates vs. protein. Well-fed flies prefer to consume sugar over yeast, a good protein source, whereas protein-deprived flies shift their preference toward yeast (Ribeiro and Dickson, 2010; Vargas et al., 2010). Protein-deprived flies also show changes in foraging behavior: they reduce global exploration and focus on visiting yeast patches in a localized area (Corrales-Carvajal et al., 2016). Two DANs projecting to the “wedge” region of the *Drosophila* brain (DA-WED cells) promote yeast consumption and suppress sugar intake after protein deprivation (Figure 2B; Liu et al., 2017). The ability of DA-WED cells to regulate yeast and sugar intake in opposing ways is due to connections with distinct postsynaptic partners on different branches of the neuron. Postsynaptic neurons projecting to the fan-shaped body and lateral accessory lobe (FB-LAL cells) promote yeast consumption, whereas postsynaptic neurons projecting to the posterior lateral protocerebrum (PLP cells) regulate sugar consumption. Protein deprivation enhances the activity of DA-WED cells as well as inducing branch-specific plasticity that increases the number of synapses with FB-LAL neurons (Liu et al., 2017). Thus, internal state induces both functional and structural plasticity of neuromodulatory cells to exert opposing effects on distinct downstream circuits, resulting in a shift from sucrose to yeast consumption.

Mated females have higher protein and salt needs than males or virgin females due to the demands of egg production. When protein-deprived, mated females shift their preference toward yeast over sucrose much sooner than males or virgin females (Ribeiro and Dickson, 2010; Vargas et al., 2010). Similarly, mating causes females to increase salt consumption and shift their preference toward higher salt concentrations (Walker et al., 2015). The post-mating shift in both yeast and salt preference relies in part on sex peptide (Ribeiro and Dickson, 2010; Walker et al., 2015), a peptide present in male seminal fluid that is transferred to the female during mating (Chen et al., 1988). Sex peptide acts on the sex peptide receptor (SPR), which is expressed in the female reproductive tract, and SPR activation represents a global post-mating signal that modulates a variety of behaviors (see below; Yapici et al., 2008; Hasemeyer et al., 2009). The post-mating shift in both yeast and salt preference is elicited even when egg production is blocked showing that it represents a feedforward modulation of nutrient intake that anticipates rather than reacts to changing nutrient needs (Ribeiro and Dickson, 2010; Walker et al., 2015, 2017). Downstream of SPR-expressing neurons, the mechanisms for modulating yeast and salt appetite diverge, since octopamine is required for enhanced preference toward yeast but not salt (Walker et al., 2015).

Interestingly, protein deprivation and mating enhance yeast preference by distinct mechanisms. This difference may arise because protein deprivation represents an urgent state of nutrient deficiency, whereas mating reflects the anticipation of increased nutrient demand in the future. Protein deprivation, but not mating, enhances the sensory responses of yeast-sensing taste neurons that drive feeding (Steck et al., 2018). A follow-up study performed functional imaging of yeast-evoked activity across the subesophageal zone (SEZ), the primary taste region of the fly brain (Münch et al., 2021). The SEZ contains the output projections of taste sensory neurons, the motor neurons that control feeding, and potentially the circuitry that connects them. Protein deprivation globally enhanced yeast-evoked activity across the SEZ and led to faster responses in motor regions. In contrast, mating had a more selective effect: it enhanced neural activity primarily in putative motor areas, an effect observed only when flies were also protein-deprived.

The modulatory effects of protein deprivation and mating can also be dissociated at the behavioral level by examining detailed metrics of foraging (Corrales-Carvajal et al., 2016). For instance, mating increases the probability that a fly will stop at a yeast patch it encounters. Amino acid deprivation does not affect this behavior in virgin flies but dramatically increases it in mated flies, revealing the synergistic effects of mating and protein deprivation. Future work will be needed to determine whether this synergy reflects convergence onto a common circuit or parallel activation of separate pathways.

Overall, we have just begun to scratch the surface in understanding how energy state and nutritional needs regulate feeding-related behaviors. Different states are typically sensed by different mechanisms, may be encoded by different neuromodulators, and may target distinct sensory or motor pathways. Even states that lead to similar behavioral changes—mating and yeast deprivation—engage distinct forms of modulation. The diversity of mechanisms capable of modulating the same neural circuits likely allows for more control and flexibility in adapting an animal’s behavior to changing internal needs.

### Emotion-Like States

Homeostatic internal states, such as those reflecting nutrient deprivation, are easily extended to model organisms such as *Drosophila*. Whether simple organisms also have emotion-like states is less clear. Anderson and Adolphs (2014) define emotion as an internal state encoded by specific patterns of neural activity that gives rise to observable behaviors. They argue that organisms as simple as flies have internal states sharing characteristics of emotion (Anderson and Adolphs, 2014). These emotion-like states are not necessarily homologous to human emotions, but they are similar in that they represent persistent internal states that modulate behavior. For example, repeated mechanical stimulation (strong air puffs) induces a state resembling arousal, in which flies show increased locomotor activity for ~10 min and remain hypersensitive to startle-inducing stimuli even after the locomotor activity has normalized (Lebestky et al., 2009). This state relies on dopamine signaling in the ellipsoid body of the central complex. A similar arousal state can be induced by repeated presentation of a moving shadow, representing a visual threat (Gibson et al., 2015).

Sexual arousal is an internal state that has been well-studied in *Drosophila* males. Male sexual behavior toward females is controlled by a set of male-specific command neurons called P1 neurons, which integrate sensory cues from females and activate motor programs for courtship (Kohatsu et al., 2011; Clowney et al., 2015). P1 neurons also promote aggression toward other males, a male-typical behavior that is evoked by the presence of a female (Hoopfer et al., 2015). In addition to acutely promoting courtship or aggression, brief optogenetic activation of P1 neurons promotes a long-lasting increase in these behaviors (Hoopfer et al., 2015; Jung et al., 2020). This has been interpreted as a state of increased sexual or social arousal. For instance, low-intensity optogenetic stimulation of P1 neurons in solitary males may evoke no overt behavioral change, but these males show increased aggression when presented with another male after the stimulation has ended (Hoopfer et al., 2015). The internal state induced by P1 activation lasts for at least 10 min and decays over time. A similar state can be triggered more naturally by exposure to a female: males are more aggressive even after the female is removed (Jung et al., 2020).

**Figure 3:**
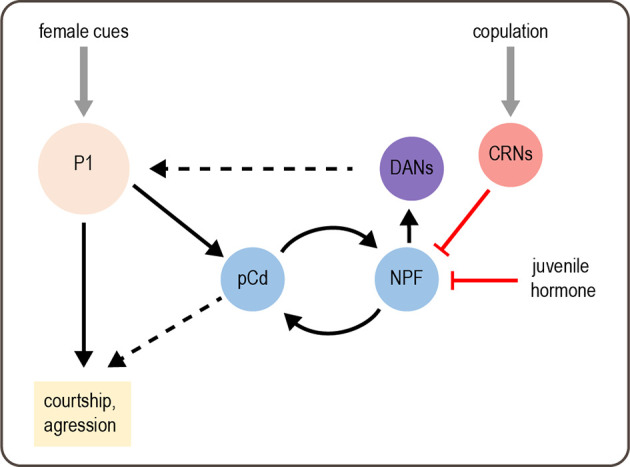
Modulation of male behavior by sexual arousal state. Female cues activate P1 neurons, which acutely promote courtship and aggression. P1 activation also elicits a state of sexual arousal that lasts for several minutes and enhances sexual behaviors. This state is mediated by persistent activity in pCd neurons. On longer timescales, a recurrent circuit comprising pCd and NPF-expressing cells maintains a sexual arousal state. Activity in this circuit builds up over days if males do not copulate, and this activity promotes mating by enhancing P1 activity *via* DANs. Copulation suppresses activity in the recurrent circuit, as does juvenile hormone circulating in young males, thus suppressing mating behavior. Gray arrows depict sensory input; solid black arrows depict neural circuit connectivity; red arrows depict inhibitory modulation. Dashed black arrows represent more complex or unknown forms of modulation: DANs enhance P1 responses by desensitizing P1 to inhibition, and the targets of pCd are unknown.

How is a persistent state of sexual arousal encoded in the brain? Although this state is induced by stimulating P1 neurons, P1 neurons do not show persistent activity following stimulation (Hoopfer et al., 2015). Thus, the sexual arousal state must be encoded by downstream neurons. An imaging screen identified a set of neurons called pCd cells that show persistent activity in response to transient P1 activation (Jung et al., 2020). The activity of pCd neurons is required for the persistent courtship and aggression elicited by transient P1 activation or exposure to a female. Activating pCd neurons amplifies courtship and aggression behaviors but cannot elicit these behaviors in the absence of an appropriate target (a female or male fly, respectively) as P1 activation does. These results suggest that a persistent state of sexual arousal is encoded by persistent activity in pCd neurons and modulates the intensity of social behaviors (Figure 3).

A separate study found that mating drive over much longer timescales, such as days, is maintained by a recurrent circuit comprising pCd neurons and NPF-expressing neurons, which excite each other (Zhang et al., 2019). This is reminiscent of recurrent excitation in other systems, which generates persistent activity underlying functions such as working memory (Wang, 2008). Activity in this recurrent circuit parallels male mating drive, which is suppressed by repeated matings and gradually recovers over several days (Zhang et al., 2016, 2019). This suppression of courtship relies on copulation and is therefore distinct from courtship conditioning, a well-characterized effect in which males reduce courtship after being rejected by pre-mated females (Griffith and Ejima, 2009). The copulation-induced “sexual satiety” state likely represents an adaptive response that prevents males from expending energy on mating when their reproductive fluids have been depleted. A set of copulation reporting neurons (CRNs) in the abdominal ganglion are responsible for inducing the sexual satiety state. CRNs project dendrites to the genitalia and axons to the brain, where they inhibit the activity of NPF neurons in the recurrent circuit to decrease mating drive (Figure 3; Zhang et al., 2019).

Recovery from sexual satiety is correlated with a gradual increase in the intrinsic excitability of pCd and NPF neurons in the recurrent circuit (Zhang et al., 2019). Neuronal excitability is in turn controlled by CREB2, an activity-dependent transcription factor representing the homolog of mammalian CREB. CREB2 modulates neuronal excitability to prolong the satiety recovery time, which ensures that it parallels the replenishment of reproductive fluids after repeated mating. CREB2 is also involved in maintaining other long-lasting states, such as circadian rhythms and memory, demonstrating a conserved function across different systems (Lonze and Ginty, 2002). Interestingly, the same cellular and circuit mechanisms used to induce sexual satiety after mating are employed to suppress courtship in another context: in juvenile males, who do not court females. Juvenile hormone released in recently enclosed males suppresses the activity of recurrent pCd and NPF neurons to reduce mating drive (Zhang S. X. et al., 2021). Thus, different states can reuse the same molecular and circuit mechanisms to induce flexible behavior.

The work described above reveals how sexual arousal and satiety states are induced and maintained. How do they actually modulate behavior? The recurrent pCd/NPF circuit promotes mating drive by enhancing dopaminergic activity in the superior medial protocerebrum (Figure 3; Zhang et al., 2019). Dopamine modulates responses of P1 neurons, which integrate excitatory and inhibitory inputs to promote courtship (Clowney et al., 2015). Specifically, dopamine de-sensitizes the P1 response to inhibition from GABAergic neurons, suggesting that dopamine may help sustain P1 excitation during courtship (Zhang et al., 2018). These results suggest that P1 neurons not only induce a sexual arousal state but also represent a target of state-dependent modulation.

Sexual arousal state also modulates visual processing to enhance a male’s ability to track a female during courtship. The underlying mechanisms have been studied using tethered males walking on a ball and tracking a moving visual stimulus representing a female (Ribeiro et al., 2018; Hindmarsh Sten et al., 2021). Increasing the male’s sexual arousal state by brief P1 activation or presentation of female pheromones caused the male to track the stimulus more closely (Hindmarsh Sten et al., 2021). This arousal state gates the activity of LC10 visual neurons, which are required for tracking a female and elicit courtship behaviors in an arousal-dependent manner (Ribeiro et al., 2018). LC10 neurons respond strongly to the moving target only when males are aroused (Hindmarsh Sten et al., 2021), revealing dynamic modulation of sensorimotor processing by sexual arousal state.

Less work has been done on sexual arousal states in female flies, although it has long been known that recently mated females are less sexually receptive (Manning, 1962). As mentioned above, the female post-mating state is induced by the activation of SPR by sex peptide, contained in the male seminal fluid (Chapman et al., 2003; Liu and Kubli, 2003; Yapici et al., 2008). Sex peptide inhibits the activity of SPR-expressing neurons in the female reproductive tract, and this mating signal is then transmitted to postsynaptic sex peptide abdominal ganglion (SAG) neurons and downstream pC1 neurons in the brain (Hasemeyer et al., 2009; Feng et al., 2014; Wang et al., 2020).

Female pC1 neurons respond to stimuli that promote mating, including male courtship song and male pheromones, and PC1 activity promotes sexual receptivity (Zhou et al., 2014). The activity of pC1 neurons is decreased by sex peptide, representing a modulatory node to regulate post-mating behaviors. pC1 neurons provide input onto descending neurons [neurons projecting from the brain to the ventral nerve cord (VNC)] called vpoDNs that control vaginal plate opening, a key component of sexual receptivity (Wang et al., 2021). vpoDNs integrate excitatory signals from pC1 neurons as well as auditory neurons tuned to the male’s courtship song. Because pC1 activity is lower after mating, this reduces the excitatory drive onto vpoDNs and leads to lower receptivity. A parallel pathway independent of sex peptide also acts to suppress female receptivity on short timescales. Abdominal neurons detect copulation, likely based on mechanosensory cues, and activate a circuit comprising ascending neurons and central peptidergic neurons that reduce sexual receptivity (Shao et al., 2019).

pC1 neurons act *via* a similar but inverse circuit mechanism to regulate the post-mating switch in egg-laying behavior. pC1 cells activate inhibitory interneurons that suppress the activity of oviDN descending neurons, which promote egg-laying (Wang et al., 2020). Decreased pC1 activity after mating causes disinhibition of oviDN activity, which leads to increased egg-laying.

The activity of pC1 neurons in females has been proposed to encode a sexual arousal state analogous to that described above for males (Wang et al., 2021). Indeed, transient activation of pC1 neurons induces persistent changes in female behavior, similar to P1 activation in males. For several minutes following pC1 activation, females are more sexually receptive (Deutsch et al., 2020). They also show other behaviors such as shoving and chasing, which may represent aggression. These behaviors appear to be driven by a separate subset of pC1 neurons from those promoting receptivity. Imaging experiments reveal that transient pC1 activation elicits persistent activity in multiple brain areas and this is likely due to recurrent connectivity (Deutsch et al., 2020). Together, studies suggest that recurrent excitation likely maintains persistent neural activity and sexual arousal states in both males and females. These states modulate sexual and social behaviors to balance a fly’s reproductive drive with other needs, such as conserving energy.

### Competing Internal States

Most studies of internal state examine the effects of one state at a time. However, animals may also experience multiple internal states that reflect competing needs. For example, hunger and thirst are different states that lead an animal to prioritize different behaviors, namely food vs. water consumption. Food consumption alleviates hunger, but by increasing blood sugar levels it increases blood osmolality and exacerbates the need for water. Flies experiencing mild starvation consume less sugar if they are also water-deprived (Jourjine et al., 2016). Four interoceptive SEZ neurons (ISNs) integrate hunger and thirst cues and regulate sugar and water consumption in opposing ways (Jourjine et al., 2016). ISNs are directly activated by AKH, a hunger signal, and their activation promotes feeding. Conversely, ISN activity is inhibited by high extracellular osmolality, which signals thirst, and their activity inhibits water consumption. Thus, a single set of neurons integrates competing internal states in order to regulate consumption behaviors and maintain homeostasis.

Another study investigated how male flies choose between feeding and mating when they are both food- and sex-deprived (Cheriyamkunnel et al., 2021). The decision to mate or feed depends on the duration of food deprivation. After 15 h of starvation, male flies prioritize feeding over courtship. Their decision is also modulated by the quality of the food as flies show less preference for feeding over mating when presented with low-calorie food. Tyramine, a biogenic amine considered to be an analog of norepinephrine, modulates distinct pathways to regulate this choice. Tyramine receptor-expressing neurons in the posterior lateral protocerebrum (TyrR^PLP^ neurons) promote feeding over courtship. Conversely, P1 neurons, previously identified as courtship command neurons, promote courtship over feeding. Tyramine acts as a satiety signal that modulates both of these pathways: tyramine inhibits the TyrR^PLP^ neurons while activating P1 neurons, thus shifting the choice toward courtship over feeding.

A general principle emerging from these studies is that the same neuromodulator (tyramine) or set of neurons (ISN cells) may be used to modulate multiple pathways in opposing ways. By simultaneously activating and inhibiting different pathways that promote competing behaviors, the balance can be shifted toward one behavior or another. This principle is also reminiscent of behavioral switches that can occur during a single internal state, such as switching from acetic acid aversion to attraction during hunger or switching from sugar to yeast preference during protein deprivation (see above). In all of these examples, competing behavioral pathways are modulated in opposing ways by internal state.

## Modulation by Behavioral State

Similar to internal states such as hunger, an animal’s behavioral state can modulate how the animal responds to stimuli in the world. For instance, the same sensory cue may have different salience or even a different meaning depending on whether a fly is walking, flying, or stationary. Like modulation by internal state, flexibility due to behavioral state can reflect modulation of sensory processing, sensorimotor transformations, or motor responses. It is important to note that changes in behavioral state are likely to correspond with changes in internal state, whether it is the internal state that alters behavioral state (e.g., increased arousal promotes locomotor activity) or *vice versa* (e.g., being active increases arousal).

### Modulation of Visual Motion Processing

**Figure 4:**
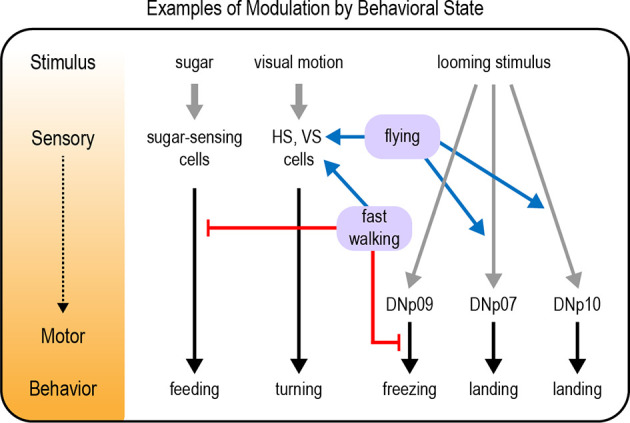
Examples of modulation by behavioral state. Behavioral states such as walking or flying modulate neural pathways underlying a variety of behavioral responses, including feeding responses, visual motion processing, and responses to a looming stimulus. Modulation can occur at any level from sensory to motor processing (represented by the diagram and color gradient on the left). Gray arrows depict sensory input; black arrows depict neural circuits that generate behavior; blue arrows depict excitatory modulation whereas red arrows depict inhibitory modulation.

Several examples of sensory modulation by behavioral state have been documented in the *Drosophila* visual system, particularly in motion-sensing pathways. These results parallel findings in the mammalian visual system (Niell and Stryker, 2010). Flies detect visual motion through horizontal (HS) and vertical (VS) neurons in the optic lobe. HS and VS cells show enhanced visual responses when flies are walking or flying, respectively (Figure 4; Chiappe et al., 2010; Maimon et al., 2010; Suver et al., 2012). This increased sensitivity could allow flies to more effectively use visual motion cues as they are actively navigating an environment (van Breugel et al., 2014). Walking also shifts the tuning of HS cells towards faster motion (Chiappe et al., 2010), a likely adaptation to the fact that the visual world moves more rapidly when flies are walking. Flying did not shift the peak of the tuning curve peak in VS cells, but it broadened tuning by enhancing responses to faster motion (Suver et al., 2012).

The HS cells receive multiple types of motor signals related to walking, which modulate the cells’ membrane potential independently of visual input (Fujiwara et al., 2017). One component of the signal encodes the general walking state, whereas other components encode the speed and direction of walking. These motor signals modulate visual responses differently depending on whether the visual motion is in the same direction as expected from the fly’s own movement. The integration of sensory and motor signals in HS cells may allow them to control walking behavior or create internal estimates of self-movement.

The enhancement of visual responses during flight or walking relies on modulation by octopamine (Suver et al., 2012; Strother et al., 2018), a mechanism also implicated in other insects (Longden and Krapp, 2010; Jung et al., 2011; Cheng and Frye, 2020). Octopamine has long been considered an analog of norepinephrine, a “fight-or-flight” signal that elicits a general state of arousal (Roeder, 2005). Flying enhances the activity of octopaminergic neurons innervating the optic lobe (Suver et al., 2012), but the circuits that elicit this activation are unknown. Although octopamine may represent a general arousal signal that enhances visual processing, the speed- and direction-selective motor signals transmitted to HS cells likely have a different source. These signals do not arise from visual or mechanosensory feedback generated during walking and may represent an internal copy of the motor command (Fujiwara et al., 2017). Similarly, motor command signals are conveyed to visual neurons during flight turns to suppress the visual response to self-motion (Kim et al., 2015, 2017). This ensures that flies do not inappropriately respond to perceived visual motion elicited by their own voluntary movement.

Overall, the modulation of visual motion processing by behavioral state likely plays several important roles in behavior. Gain changes may increase the salience of visual cues when the fly is walking or flying, whereas tuning changes ensure that moving flies detect motion at faster speeds. Speed- and direction-selective signals may allow flies to cancel out self-generated visual responses or use them to control their movement.

### Modulation of Other Visually-Guided Responses

A fly’s behavioral state also alters visual responses beyond motion-sensing. Positive phototaxis, or preference for light, is a well-studied innate behavior in *Drosophila* (Benzer, 1967). Interestingly, eliminating a fly’s ability to fly by clipping or gluing the wings abolishes positive phototaxis and can switch its behavior to light aversion, even though this assay does not involve flight (McEwen, 1918; Gorostiza et al., 2016). This effect is not induced by damage to other organs (Gorostiza et al., 2016) and may have evolved because flightless flies are highly vulnerable to predators in the light. Dopaminergic and octopaminergic neurons were implicated in mediating photopreference (Gorostiza et al., 2016), but the mechanisms underlying the behavioral switch are still unknown. Presumably, flies must continuously monitor their ability to fly and modulate their visual responses accordingly.

Responses to looming visual stimuli are strongly modulated by behavioral state. Looming stimuli represent the approach of an object, often a predator. Flies respond to looming stimuli with defensive behaviors such as jumping, freezing, fleeing, or flight takeoff (Card and Dickinson, 2008; von Reyn et al., 2014; Zacarias et al., 2018). The response that a fly chooses depends on its behavioral state. Flies that are walking more slowly at the time of the threat are more likely to freeze than flee (Zacarias et al., 2018). This may be an adaptive response reflecting the fact that slowly moving flies cannot accelerate quickly enough to escape, so freezing is the better option. A pair of descending neurons, the DNp09 neurons, are required for freezing but not fleeing, demonstrating that these responses are mediated by distinct motor pathways (Zacarias et al., 2018). Optogenetic activation of DNp09 neurons causes flies to freeze, and the probability of freezing is higher if the flies are walking more slowly before stimulation. These results imply that information about walking speed is integrated downstream of DNp09 neurons (Figure 4), perhaps by gating motor neuron activation in the VNC.

Although a looming stimulus may represent the approach of a predator, this visual pattern also occurs when a moving fly intentionally approaches an object. In this case, a defensive response would be inappropriate. Indeed, the same looming stimulus that evokes escape behavior in a standing fly elicits a landing response in flying flies (Ache et al., 2019a). Two sets of descending neurons that promote landing responses have been identified, DNp07 and DNp10 (Ache et al., 2019a). Optogenetic activation of these neurons elicits landing-like motor responses even if the fly is not flying, revealing that the behavioral state acts upstream of these neurons to modulate their activity (Figure 4). Indeed, the visual responses of DNp07 and DNp10 are gated by state: they respond robustly to features of looming stimuli only during flight (Ache et al., 2019a). Thus, behavioral flexibility is achieved by modulating the sensorimotor transformation: visual input is coupled to the landing motor pathways only during flight.

### Modulation of Carbon Dioxide Responses

The behavioral state also modulates responses to other sensory stimuli, beyond visual cues. One prominent example is carbon dioxide. Carbon dioxide is released by animals during respiration and is emitted at three- to four-fold higher concentrations by flies when they are stressed (Suh et al., 2004). In a two-choice assay, flies robustly avoid the chamber containing carbon dioxide, suggesting that they may interpret carbon dioxide as a stress signal (Suh et al., 2004). However, flies switch their behavioral response from aversion to attraction when they are active, such as walking at high speeds or flying (Wasserman et al., 2013; van Breugel et al., 2018). Since carbon dioxide is emitted by fermenting fruit, attraction to carbon dioxide may help guide flies to their preferred food source. The aversive and attractive responses to carbon dioxide are mediated by separate olfactory receptors and neural pathways (Wasserman et al., 2013; van Breugel et al., 2018). Octopamine is required for flying flies to show carbon dioxide attraction (Wasserman et al., 2013), suggesting that neuromodulation may alter the gain of one or both of the olfactory pathways to shift the balance toward attraction—potentially similar to how hunger switches acetic acid aversion to attraction, as described above.

### Other Examples of Modulation by Behavioral State

As described above, walking state modulates diverse responses such as visual motion processing, responses to looming stimuli, and the response to carbon dioxide. Indeed, locomotor activity seems to be one of the most critical behavioral states an organism needs to account for. Large-scale imaging studies have found that walking state increases global brain activity, suggesting that this state signal is transmitted to many different circuits with different functions (Aimon et al., 2019; Schaffer et al., 2021). One function of this state-dependent modulation may be to suppress behaviors that should not be expressed during walking. For example, flies must be stationary in order to feed on a substrate. Walking suppresses the initiation of feeding, and this is mediated by interneurons that are activated by mechanosensory inputs from the legs (Figure 4; Mann et al., 2013). Conversely, feeding seems to suppress locomotion: walking is reduced if the proboscis is maintained in an extended position, as occurs during feeding (Mann et al., 2013). The inhibition of one behavior during another is likely to be a general example of how behavioral state modulates neural processing.

Cande et al. (2018) took an unbiased approach to examine how a fly’s current behavioral state modulates the behavioral responses induced by optogenetically activating subsets of descending neurons. They used an unsupervised method to characterize a variety of behaviors, such as locomotion, body movements, and grooming and found that behavioral changes elicited by optogenetic stimulation depended on what the fly was doing just before stimulation. In some cases, neuronal activation could induce different behaviors on different trials, and the fly’s behavior before stimulation was highly predictive of which behavior was elicited by activation. These state-dependent effects reflect modulation downstream of the descending neurons, suggesting that significant modulation and processing occurs in the VNC.

Another study used a different unbiased approach to identify states that influence how males produce courtship song (Calhoun et al., 2019). Males modulate their song production depending on the female’s distance, orientation, and movement as well as their own movement. However, the influence of these cues on song production varies depending on the male’s state. Three states were identified using an unsupervised “GLM-HMM” approach and correspond roughly to periods when the male is chasing the female, close to the female without chasing, or residing far from the female and oriented away from her. The male is unlikely to sing in the latter state, whereas the former two states generate different types of song. Specific sensory cues, such as the male’s velocity or female’s distance, are weighted differently in different states in determining song production. This study reveals the power of unbiased behavioral analysis in identifying states that modulate the mapping of sensory input to behavior. Although the states identified in this study correspond to different behavioral states, such as the male chasing the female, this need not be the case.

Overall, behavioral states can modulate neural circuits at any level: from sensory processing to sensorimotor transformations to motor pathways. This is reminiscent of modulation by internal states such as hunger. Behavioral states can also be detected in multiple ways, such as by sensory feedback or an internal copy of the motor command. Ultimately this modulation may alter the likelihood of expressing a behavior or switch the behavioral response entirely.

## Modulation by Environmental Context

Thus far we have discussed how a fly’s state, either an internal state or an ongoing behavioral state, modulates its behavioral responses and actions. In addition, a fly needs to continuously evaluate the environmental context in which stimuli are encountered. Context may be conveyed by the specific features of a stimulus or the presence of additional cues that modulate the response to a given stimulus.

### Flexibility in the Response to a Single Stimulus

The response to a single stimulus can vary based on features of the stimulus that reflect its context. One example is the escape response to looming stimuli. Responses to looming stimuli are modulated by behavioral state, as described above, but also show additional flexibility. In a paradigm in which looming stimuli elicit flight takeoff, flies select between two takeoff modes: short and long (von Reyn et al., 2014). The short takeoff is faster but less controlled: flies trade off stability for speed. The same stimulus can evoke different takeoff modes on different trials, but flies choose the short takeoff more often if the stimulus is approaching faster.

The short and long takeoff modes are generated by different motor pathways. The giant fiber neurons, a pair of large descending neurons, mediate short but not long takeoffs (von Reyn et al., 2014). The giant fiber neuron is activated during both takeoff modes but spikes more quickly during short takeoff trials, suggesting a timing-based model: the motor pathway that is activated first determines the type of takeoff. The two motor pathways are differentially sensitive to specific features of the looming stimulus, including looming size and velocity, and these different features are processed in separate visual input pathways (von Reyn et al., 2017; Ache et al., 2019b). By integrating specific features of the stimulus and activating the appropriate motor pathway, flies can bias their behavior to maximize their chance of escape.

### Context-Dependent Modulation of Carbon Dioxide Responses

The response to a stimulus can also vary based on the presence or absence of other cues within the environment. Previously we discussed how flies change their response to carbon dioxide depending on their behavioral state, and this may reflect a balance between the role of carbon dioxide as a potential distress signal as well as a product of fruit fermentation. In addition to behavioral state-dependent modulation, flies modulate their response to carbon dioxide depending on the presence of other olfactory cues. Flies display robust aversion to carbon dioxide in a T-maze (Suh et al., 2004), free-flying two-trap choice assay (Faucher et al., 2013), and four-field olfactometer assays (Faucher et al., 2006). Despite this innate aversion, when vinegar is present a starved fly significantly reduces its avoidance to carbon dioxide (Bracker et al., 2013; Lewis et al., 2015). Further, vinegar mixed with aversive concentrations of carbon dioxide is more appetitive to free-flying flies than vinegar alone (Faucher et al., 2013). In these instances, the fly appears to prioritize seeking food over avoiding carbon dioxide.

**Figure 5:**
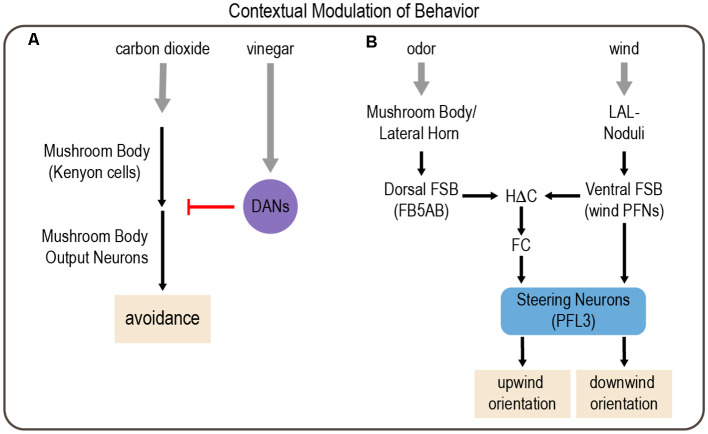
Examples of modulation of behavior by environmental context involving integration of multiple sensory cues. **(A)** Context-dependent responses to carbon dioxide depend on the mushroom body (MB). Kenyon cells (KCs) in the MB are activated by carbon dioxide, which activates MB output neurons (MBONs) to drive avoidance responses. However, the presence of vinegar activates subsets of DANs, which inhibit MBON activation to reduce carbon dioxide avoidance. **(B)** Odor and wind cues are processed by parallel pathways that are integrated in the FSB to drive wind orientation. *Drosophila* typically orient downwind from a wind source. However, in the context of an appetitive odor cue, this behavior reverses, and flies orient upwind. Gray arrows depict sensory input; black arrows depict neural circuit connectivity; red arrow depicts inhibitory modulation.

The MB, previously discussed as an integration center for hunger and satiety cues, appears to mediate the context-dependent modulation of carbon dioxide avoidance (Bracker et al., 2013). Initial studies revealed that MB inactivation during the presentation of carbon dioxide along with vinegar switches the response from aversion to attraction in both fed and starved flies. A follow-up study identified a specific set of glutamatergic MBONs that are activated by carbon dioxide and promote avoidance, and their response is diminished by the addition of vinegar (Lewis et al., 2015). This modulation is mediated by a set of DANs that innervate the same MB regions as the MBONs and likely act presynaptically. Optogenetic activation of these DANs suppresses carbon dioxide avoidance, and imaging data demonstrate that these DANs are strongly and specifically activated by vinegar. Previous work suggested that dopaminergic activity negatively regulates carbon dioxide avoidance (Siju et al., 2014), but this was the first study to identify specific DANs that play a critical role (Lewis et al., 2015). Together these data suggest a circuit mechanism in which glutamatergic MBONs promote carbon dioxide avoidance and this avoidance is suppressed in the presence of vinegar by activating DANs that inhibit MBON activity (Figure 5A). Interestingly, the suppression of carbon dioxide avoidance is not long-lasting. Thus, vinegar and the resulting DAN activation briefly inhibit carbon dioxide avoidance in an effort to prioritize food-seeking behaviors.

### Cross-Modal Modulation

The behavioral response to a stimulus can also be modulated by sensory integration across multiple modalities. For instance, although feeding responses are primarily determined by the taste of a food source, they can be strongly modulated by its smell, texture, and temperature. Flies show a feeding response when their legs contact sugar and the presence of yeast odor enhances this response by acting through OR35a-expressing OSNs (Oh et al., 2021). Robust feeding responses also require mechanosensory input from the leg, which provides information related to the viscosity of the food. Both smell and touch work synergistically to enhance feeding responses elicited by sugar, enabling flexible feeding behavior based on multiple sensory properties of the food (Oh et al., 2021).

Other work demonstrates the importance of temperature in modulating taste perception and food preference. Like other animals, *Drosophila* show reduced taste attraction to sugar at lower temperatures (Li Q. et al., 2020). The lower preference for sucrose at cool temperatures persists despite starvation. The ethological relevance of this effect has not been demonstrated, but one possibility is that fresh fruit may be cooler than decaying fruit, which flies prefer. The reduction in sugar attraction could result from reduced activation of sugar-sensing neurons at cool temperatures. However, imaging and electrophysiological data suggest otherwise. Instead of suppressing sugar-sensing gustatory neurons, cool temperatures activate aversive bitter-sensing neurons and mechanosensory neurons to ultimately drive a decrease in feeding responses (Li Q. et al., 2020). Thus, feeding responses at cool temperatures are guided by the simultaneous activation of competing sensory pathways. This mechanism resembles the mechanism underlying the feeding response to acetic acid, described above, which is also determined by the balance between activation of the sugar- and bitter-sensing pathways (Devineni et al., 2019).

Cross-modal modulation of behavior also occurs while flies are in flight. Flies steer toward elongated vertical bars that visually resemble feeding sites, such as vegetation, but they turn away from aversive wind sources (Maimon et al., 2008). When aversive wind currents arise from the same location as the attractive visual cue, flies display a dynamic response in which they initially turn away and then slowly turn back toward the cue (Currier and Nagel, 2018). The aversive mechanosensory information arises, at least in part, from the antenna. Modeling studies suggest that visual and mechanosensory signals are independently processed to generate modality-specific turn commands. These signals converge and summate to ultimately drive turning behavior (Currier and Nagel, 2018).

Flies also use cross-modal integration to orient upwind in the presence of an attractive odor cue, which would enable them to navigate toward an odor source. Odor and wind cues are integrated in a region of the central complex called the fan-shaped body (FSB; Matheson et al., 2021). Output neurons from the MB and lateral horn, the two higher-order olfactory processing centers (Marin et al., 2002; Su et al., 2009), converge on neurons that provide input to the dorsal FSB. A separate class of FSB neurons, hΔC neurons, integrate odor information from the dorsal FSB and wind direction cues from the ventral FSB. In the absence of odor, flies tend to orient downwind, suggesting that competing pathways operate in the presence and absence of odor. Circuit analysis and computational modeling suggest that a direct pathway from wind-activated FSB neurons to steering FSB output neurons (PFL3 cells) normally promotes downwind orientation, but the presence of an attractive odor activates an indirect pathway comprising hΔC neurons, which alters PFL3 activation to promote upwind orientation (Figure 5B; Matheson et al., 2021).

In contrast to elongated vertical bars, which flies steer toward, flies steer away from smaller objects, which are presumably interpreted as a threat (Maimon et al., 2008). This avoidance of small objects switches to attraction in the presence of an attractive odor, such as vinegar (Cheng et al., 2019). The presence of a food odor may signify that the small object is not a threat but in fact a potential food source. This odor-dependent switch from visual aversion to attraction can be mimicked by optogenetically activating modulatory octopaminergic neurons or motion-sensitive visual pathways in the optic lobe (Cheng et al., 2019). Previous work showed that vinegar activates octopaminergic neurons innervating the visual system (Wasserman et al., 2015). Cheng et al. (2019) propose a model whereby two different visual pathways compete to determine behavior: a motion vision pathway that drives approach and an object detection pathway that drives avoidance. An appetitive odor activates octopaminergic neurons, which enhances the gain of the motion vision pathway and tips the balance towards approach. As discussed above, flight itself recruits octopaminergic enhancement of visual motion responses (Suver et al., 2012). The additional role of octopamine in mediating odor-dependent modulation suggests that octopamine plays multiple roles in visual processing and its different effects can be superimposed.

Thus, the integration of different environmental cues is a common way in which behavioral responses exhibit flexibility. Classic studies have provided significant insight into sensory processing by presenting a single stimulus at a time, but paradigms with multiple stimuli will likely reveal far greater complexity in behavioral responses and the underlying neural circuitry. Moreover, how different cues are integrated and the behavioral responses they elicit are likely to differ across *Drosophila* species depending on the ecological niches that they occupy.

## Behavioral Flexibility in Learning and Memory

Learning can be defined as an experience-dependent change in behavior. Storing past experiences as memories allows animals to apply learned information when faced with a similar situation. Thus, learning and memory is a prime example of flexible behavior. A wealth of studies investigating learning and memory in the fruit fly have provided significant insight into the underlying neural networks and how they are modified over different timescales. In particular, the MB has received considerable attention (Cognigni et al., 2018; Modi et al., 2020). The MB consists of densely packed Kenyon cell (KC) fibers, which receive processed olfactory, visual, gustatory, and tactile sensory input (Marin et al., 2002, 2020; Liu et al., 2012, 2016; Caron et al., 2013; Gruntman and Turner, 2013; Vogt et al., 2014, 2016; Kirkhart and Scott, 2015; Yagi et al., 2016; Zheng et al., 2018; Li J. et al., 2020). The MB, therefore, serves as a multisensory integration hub for learning as well as other behaviors, as described above. Specific KCs synapse on distinct sets of GABAergic, cholinergic, and glutamatergic MBONs (Tanaka et al., 2008; Aso et al., 2014a). Several MBONs promote either odor-driven approach or avoidance, and the balance between these pathways seems to determine the fly’s behavioral response to the odor (Aso et al., 2014b; Owald et al., 2015; Perisse et al., 2016). DANs innervating the MB encode valence-related signals and modulate the synapses between KCs and MBONs (Schwaerzel et al., 2003; Claridge-Chang et al., 2009; Aso et al., 2010; Sejourne et al., 2011; Burke et al., 2012; Liu et al., 2012; Hige et al., 2015). The MB is organized into distinct anatomical and functional modules, termed compartments (Aso et al., 2014a). Each compartment contains a defined subset of DAN inputs and MBON outputs, enabling specific valence signals to modulate specific output pathways and operate using distinct learning rules (Pai et al., 2013; Placais et al., 2013; Bouzaiane et al., 2015; Hige et al., 2015; Aso and Rubin, 2016; Perisse et al., 2016).

**Figure 6:**
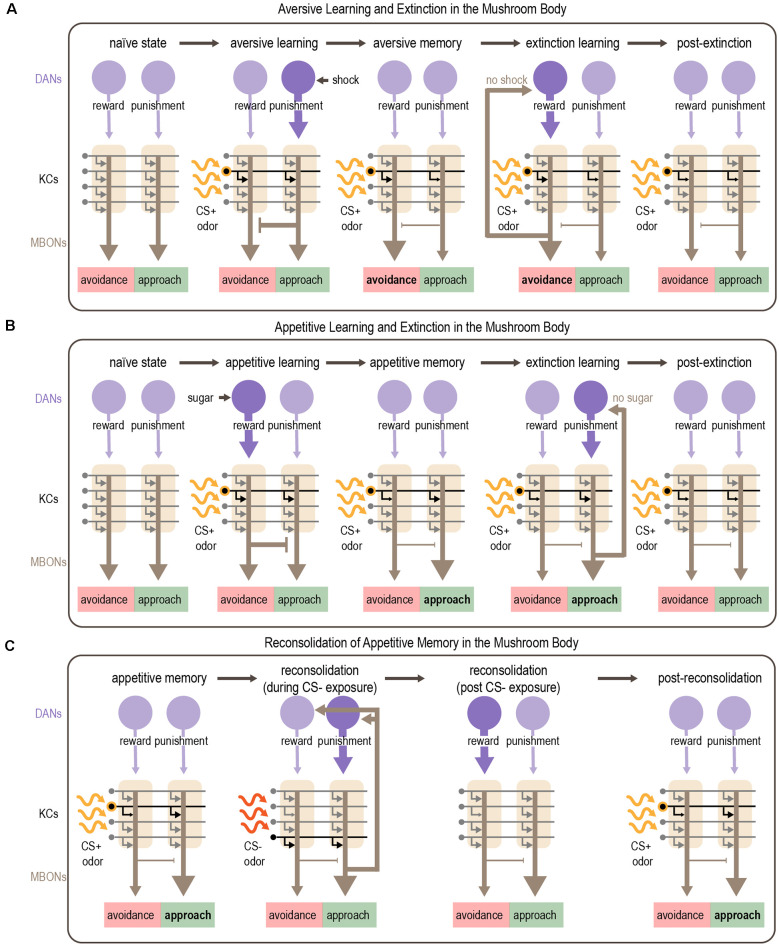
Mechanism for associative learning, extinction learning, and reconsolidation in the MB. **(A,B)** Plasticity in MB circuits mediates aversive **(A)** and appetitive **(B)** learning. Odor sparsely activates KCs within the MB. Aversive cues activate punishment-encoding DANs, which modulate MBONs promoting approach, whereas appetitive cues activate reward-encoding DANs, which modulate MBONs promoting avoidance. Dopamine depresses active KC-MBON synapses. After aversive learning, this depression shifts the balance of MBON activity towards avoidance **(A)** whereas after appetitive learning the balance shifts towards approach **(B)**. Extinction of aversive or appetitive memories occurs by readjusting the balance of MBON activity. After aversive learning, presenting the CS+ in the absence of anticipated electric shock causes avoidance-promoting MBONs to recurrently activate reward-encoding DANs, which encode a competing appetitive memory that reduces avoidance **(A)**. After appetitive learning, presenting the CS+ in the absence of anticipated sugar reward causes approach-promoting MBONs to recurrently activate punishment-encoding DANs, resulting in the formation of a competing aversive memory that reduces approach **(B)**. **(C)** Appetitive memories can be re-activated by exposure to the CS- which induces reconsolidation of the original memory. Reconsolidation requires recurrent DAN activation orchestrated by subsets of MBONs (MBON γ2α′1). Activation of punishment-encoding DANs during CS- exposure and subsequent activation of rewarding-encoding DANs after CS- exposure results in reconsolidation of the original memory, although the exact mechanisms are unclear. Purple arrows depict DAN input to the MB; gray arrows depict KC axons innervating MBONs and are shown in black when activated by odor; brown arrows depict MBON output. Note that the middle panels of **(C)** show skewed MBON output as it would be elicited by the CS+, representing the CS+ memory; output in response to the CS- or in the absence of odor is not skewed.

Although its function is complicated, the canonical view on associative learning in the MB is that different sets of DANs encode reward and punishment, and they mediate appetitive or aversive learning, respectively (Figure 6; Claridge-Chang et al., 2009; Aso et al., 2010, 2012; Burke et al., 2012; Liu et al., 2012; Waddell, 2013; Huetteroth et al., 2015; Yamagata et al., 2015). In general, reward-encoding DANs innervate compartments containing MBONs that promote avoidance, whereas punishment-encoding DANs innervate compartments containing MBONs that promote approach. Pairing an odor with a reward or punishment causes the odor-responsive KCs to be activated at the same time as the relevant DANs. The coincident activation of KCs and DANs in specific compartments typically induces synaptic depression of KC-MBON synapses within those compartments. Appetitive learning (associating odor with reward) reduces the activity of MBONs that promote avoidance, thereby skewing the network toward approach behavior (Owald and Waddell, 2015; Owald et al., 2015). Conversely, aversive learning (pairing odor with punishment, such as electric shock) reduces the activity of MBONs that promote approach, biasing the behavioral response towards avoidance (Sejourne et al., 2011; Aso et al., 2014b; Perisse et al., 2016; Felsenberg et al., 2018; McCurdy et al., 2021). Thus, memories are encoded by shifting the balance of the MB output network to bias the behavioral response and drive goal-directed behaviors (Owald and Waddell, 2015).

There are a number of studies and comprehensive reviews that discuss the synaptic and circuit mechanisms for encoding and retrieving memory (Zars, 2000; Heisenberg, 2003; Davis, 2011; Kahsai and Zars, 2011; Owald and Waddell, 2015; Cognigni et al., 2018; Boto et al., 2020; Modi et al., 2020). However, in a natural setting, associations between stimuli can quickly change, thus it is essential for animals to remain flexible and modify learned responses appropriately (Felsenberg, 2021). Here we will focus on how memories are modulated and updated, enabling behavioral flexibility beyond simple associative learning. In addition to the feedforward MB circuit organization described above, extensive feedback connections between MBONs and DANs also exist, both within and across MB compartments (Aso et al., 2014a; Li F. et al., 2020). Further anatomical work suggests that different MBONs are interconnected (Perisse et al., 2016; Felsenberg et al., 2018; Li F. et al., 2020; Scaplen et al., 2021). These lateral and recurrent connections provide a substrate to encode more complex relationships between stimuli, incorporate new information, update memories, and guide behavior.

### Extinction

During associative learning, a stimulus acquires predictive value as it is associated with punishment or reward. When stimuli are no longer predictive, it is no longer adaptive to continue to avoid or approach the conditioned stimulus (CS+). In this case, the learned response is suppressed *via* extinction learning. Memory extinction refers to the decrease in the behavioral response to the CS+ when it is no longer paired with reward or punishment. A number of studies have described extinction learning in *Drosophila* in the context of aversive (Tempel et al., 1983; Tully and Quinn, 1985; Schwaerzel et al., 2002; Lagasse et al., 2009; Qin et al., 2012; Hirano et al., 2016; Felsenberg et al., 2018) and appetitive (Felsenberg et al., 2017; Wang et al., 2019) memories.

Early work suggested that extinction relies on distinct transcriptional and molecular signaling pathways from those that underlie initial associative learning (Qin and Dubnau, 2010; Hirano et al., 2016). Although it is widely accepted that extinguishing a behavioral response does not erase the original memory trace (Bouton, 2002; Eisenhardt and Menzel, 2007; Myers and Davis, 2007; Quirk and Mueller, 2008), until recently the circuitry mechanisms supporting this process remained unclear.

Felsenberg et al. (2017, 2018) describe a circuit basis for extinction learning in *Drosophila* (Figures 6A,B). They show that when a CS+ that was previously associated with punishment is no longer predictive, extinction learning forms a parallel, competing memory for the CS+ that engages the same circuitry used for appetitive learning. Inactivation of DANs involved in reward processing prevents the formation of this competing appetitive memory and extinction cannot occur (Felsenberg et al., 2018). However, a recent study showed that sugar reward learning and the extinction of aversive memory rely on different subsets of reward-encoding DANs that innervate the same compartment (Otto et al., 2020). An analogous circuit mechanism underlies the extinction of appetitive memories, in which an aversive CS+ memory competes with the original appetitive CS+ memory (Felsenberg et al., 2017, 2018). These studies also implicated feedback connections from MBONs to DANs in mediating extinction. A recent study tested a computational model of the *Drosophila* MB that includes separate reward and punishment pathways modulated by recurrence and mutual inhibition. This model reproduced the experimental results from Felsenberg et al. (2017, 2018) suggesting that these circuit motifs are important for mediating extinction (Springer and Nawrot, 2021).

A similar circuitry mechanism for extinction, also involving dopamine, has been described in rodents (Luo et al., 2018; Salinas-Hernandez et al., 2018). This suggests a general neural framework for extinction learning across species whereby the omission of a punishment is encoded as reward and the omission of reward is encoded as punishment. These newly formed CS+ memories compete with the original CS+ memory, resulting in the neutralization of a behavioral response.

The circadian system is a well-known regulator of learning and memory (Eckel-Mahan et al., 2008; Lyons and Roman, 2009; Gerstner and Yin, 2010; Le Glou et al., 2012; Smarr et al., 2014; Krzeptowski et al., 2018; Flyer-Adams et al., 2020; Inami et al., 2020) and also plays a role in the extinction of long-term memories (Pace-Schott et al., 2015; Sun et al., 2019; Zhang Y. et al., 2021). In *Drosophila*, a network of approximately 150 clock neurons orchestrates daily rhythms in physiology and behavior (Nitabach and Taghert, 2008). Recent evidence suggests that the activity of a subset of dorsal clock neurons is required for the extinction of long-term appetitive memories (Zhang Y. et al., 2021). Specifically, the inactivation of subsets of cryptochrome-positive dorsal neurons (DN1s) and downstream neurons expressing the peptide SIFamide disrupts extinction 24 h after initial learning. Further, artificial activation of DN1s potentiated extinction learning. Calcium imaging revealed that DN1 activity increased during extinction trials. DN1s promote sleep by acting on the circadian pacemaker cells (Guo et al., 2016), suggesting a connection between sleep, circadian rhythms, and extinction. It is not clear how clock neurons and their downstream targets interact with competing neural circuits within the MB, an important area for future study.

### Reversal Learning

Reversal learning represents another example of behavioral flexibility in response to changes in expected contingencies (Jones and Mishkin, 1972). In a typical reversal learning paradigm, the animal first learns that stimulus A (CS+) predicts reward or punishment while stimulus B (CS-) does not. After learning, the stimulus-outcome contingencies are reversed: now stimulus B predicts the reward or punishment and stimulus A does not. Extinction may play a role in reversal learning as behavioral responses to the CS+ are diminished. However, reversal learning is more cognitively demanding because the reward or punishment is not merely absent but instead occurs with another stimulus that must now acquire predictive value (Izquierdo and Jentsch, 2012; Nilsson et al., 2015; Izquierdo et al., 2017). Thus, reversal learning is often considered the gold standard for assessing cognitive flexibility and, as such, is often disrupted in individuals suffering from neuropsychiatric disorders (Waltz and Gold, 2007; Murray et al., 2008; Leeson et al., 2009; Izquierdo and Jentsch, 2012; Gruner and Pittenger, 2017).

Early work in *Drosophila* highlighted the requirement of a GABAergic neuron that broadly innervates the MB for both olfactory and visual reversal learning, suggesting that it may inhibit the learned response in order to allow for new associations (Ren et al., 2012; Wu et al., 2012). The notion that reversal learning requires inhibition of the learned response has been discussed in the context of human behavior and mammalian models (Izquierdo and Jentsch, 2012). Other work in flies has highlighted the role and timing of dopamine activation in encoding memory and its reversal (Aso and Rubin, 2016; Berry et al., 2018; Handler et al., 2019).

How does reversal learning suppress the original memory? McCurdy et al. (2021) investigated this question using reversal learning in an aversive conditioning paradigm. During reversal trials when the original CS+ was no longer associated with electric shock, the activity of subsets of reward-encoding DANs increased, as also occurs during aversive extinction learning (Felsenberg et al., 2018; McCurdy et al., 2021). These DANs are activated through a recurrent circuit: the activity of punishment-encoding DANs is decreased when the expected shock is omitted, which increases the activity of a postsynaptic MBON that engages the reward-encoding DAN. Thus, a complex multi-compartment relay system is engaged to decrease CS+ avoidance and allow new associations to be learned.

Recent work has also highlighted the importance of nitric oxide, which is co-released with dopamine by DANs, in the rapid updating of memories (Aso et al., 2019). Nitric oxide acts antagonistically to dopamine to limit memory retention and enable the updating of memories in response to changing conditions. In optogenetic learning paradigms, impairing nitric oxide signaling led to longer memory retention and slower reversal learning. Nitric oxide acts in only a subset of MB compartments, enabling some MB pathways to form labile memories while other parallel MB pathways display more stable memory storage.

### Reconsolidation

Considerable evidence suggests that the mere retrieval of a memory can cause the original memory trace to become labile and vulnerable to disruption or modification (Lee et al., 2017). As a consequence, a process called post-retrieval reconsolidation is required in order to re-stabilize the memory trace. In a changing environment, reconsolidation likely provides an opportunity to incorporate new information and update a memory to maintain its relevance.

Similar to rodents (Nader et al., 2000), early work in flies demonstrated that aversive memory reconsolidation requires protein synthesis (Lagasse et al., 2009). After training, the ability to reactivate a memory was significantly reduced following treatment with protein synthesis inhibitors (Lagasse et al., 2009). More recent work identified the MB as a central brain region for reconsolidation (Felsenberg et al., 2017). In this study, appetitive learning was induced by pairing sugar with one odor (CS+) whereas another odor (CS-) was separately presented without reward, as a control. After learning, memory reactivation was triggered by presenting the CS-. Inactivation of specific DANs during CS- re-exposure disrupted reconsolidation, resulting in an impaired appetitive memory for the CS+. Interestingly, the MB compartments required for reconsolidation of appetitive memory are different from those involved in initial learning: in fact, reconsolidation engages circuits used for aversive learning. Imaging studies showed that the MBONs required for reconsolidation increased their response to the CS- after learning (Felsenberg et al., 2017). These data suggest a circuit framework in which a previously-stored appetitive memory is reactivated upon exposure to the CS-, which activates specific MBONs. These MBONs activate recurrently connected DANs, including reward-encoding DANs that reinforce the original appetitive association (Figure 6C). If this circuit is disrupted while the memory is still labile, the memory is lost.

### Latent Learning

Thus far we have discussed how newly acquired information can modify memory-related circuits after learning to alter behavior. However, information acquired before associative conditioning can also modify subsequent learning. This can occur simply by exposure to an environment or stimulus in the absence of obvious reinforcement or motivation, known as latent learning. In one form of latent learning, called latent inhibition, pre-exposure to a stimulus diminishes its ability to acquire meaning at a later time (Lubow and Moore, 1959). Suppressing the ability to learn about irrelevant stimuli is likely advantageous as it allows an animal to focus its attention on relevant stimuli.

Latent inhibition has recently been reported for the first time in *Drosophila*. Jacob et al. (2021) showed that pre-exposure to the CS+ impairs the expression of subsequently learned appetitive memories. Interestingly pre-exposure to the CS+ did not impair the expression of subsequently learned aversive memories and in fact enhanced it. Pre-exposure to the CS+ creates an aversive memory that decays within several hours, which competes with newly formed appetitive memories but facilitates aversive memories. The aversive latent memory depends on punishment-encoding DANs and MB circuitry involved in long-term aversive learning (Aso and Rubin, 2016; Aso et al., 2019). Temporally controlled neuronal silencing experiments suggest that latent inhibition results from competing aversive and appetitive memory circuits interacting during memory retrieval, and not a disruption of memory acquisition. Thus, latent inhibition, like extinction, involves the formation of parallel memories for the same stimulus, which compete to drive behavioral choice. Competing memory traces are also thought to underlie these processes in mammals (Barad et al., 2004; Lingawi et al., 2017).

### State-Dependent Flexibility in Learning and Memory

A fly’s internal state, such as hunger or thirst, can have a profound impact on learning and memory (Krashes et al., 2009; Lin et al., 2014; Senapati et al., 2019). For example, flies need to be motivated by hunger in order to express a previously formed memory for sugar (Krashes and Waddell, 2008). This requires the hunger signal NPF and the expression of its receptor in subsets of DANs innervating the MB (Krashes et al., 2009).

More recent work revealed that the presence of food after learning an odor-sugar association determines whether or not sleep is required for the consolidation of long-term memory (Chouhan et al., 2021). When flies are starved after training, sleep is not required for memory consolidation. In contrast, when flies are fed after training, sleep is essential: sleep-deprived flies show significant reductions in the expression of sucrose memory 24 h after training. Whether sleep is required for consolidation was not dependent on caloric intake, but instead depends on the hunger signal NPF. Memory consolidation in starved flies that lacked either NPF or the NPF receptor was sleep-dependent. Strikingly, the consolidation of memories in these different contexts was mediated by distinct, non-overlapping MB neural circuits. Thus, experience and context can determine which neural circuits are required to consolidate the same memory.

## Discussion

In this review, we have highlighted examples of behavioral flexibility in *Drosophila* that are related to changes in internal or behavioral states, environmental context, or learning and memory. We note that there are many other examples not discussed here, such as behavioral flexibility in the context of social experience, circadian rhythms, and sleep (Allada and Chung, 2010; Kim et al., 2018; Beckwith and French, 2019; Bentzur et al., 2021; Li et al., 2021). We also have not discussed maladaptive forms of behavioral flexibility, such as addiction (Bainton et al., 2000; Devineni and Heberlein, 2009; Kaun et al., 2011; Landayan and Wolf, 2015; Lowenstein and Velazquez-Ulloa, 2018; May et al., 2019; Scaplen et al., 2020).

Nearly all forms of behavioral flexibility rely on the same principles: the state or context must be sensed and encoded, conveyed to relevant circuits, and must then modulate neural activity within those circuits to alter the behavioral output. Few examples exist where mechanisms for all of these processes have been identified. One of the most comprehensive examples identified is the circuit suppressing male sexual drive after copulation. Studies described above have identified the neurons sensing copulation and relaying this state to the brain (CRNs), the neurons that encode sexual arousal state (recurrently connected NPF/pCd neurons), how the activity of state-encoding neurons is regulated over long timescales (CREB2 modulation of neuronal excitability), and how these neurons modulate circuits for behavior (dopaminergic modulation of P1 neurons). In the future, we expect to see more examples detailing comprehensive circuit mechanisms underlying behavioral flexibility.

Studies of behavioral flexibility in *Drosophila* reveal another general principle: circuits can be modulated at different levels, from sensory responses to motor pathways. Why might modulation at a specific level be advantageous? Modulating sensory responses early in the circuit, such as the sensory neurons themselves, would result in global modulation of all behaviors elicited by that cue. This could be advantageous if it is critical for an animal to perceive a cue as more or less salient, such as hunger-dependent enhancement of sugar sensitivity, but it does not allow for control over which behaviors are modulated. In contrast, modulating motor or pre-motor pathways would enable an internal state to gate the activation of specific behaviors or motor programs. Some forms of behavioral state-dependent modulation occur downstream of descending neurons and are likely to represent modulation of motor pathways. State-dependent modulation can also act in between sensory and motor pathways to modulate the sensorimotor transformation. Examples of this include the arousal-dependent gating of courtship pursuit and flight-dependent responses to looming stimuli.

**Figure 7:**
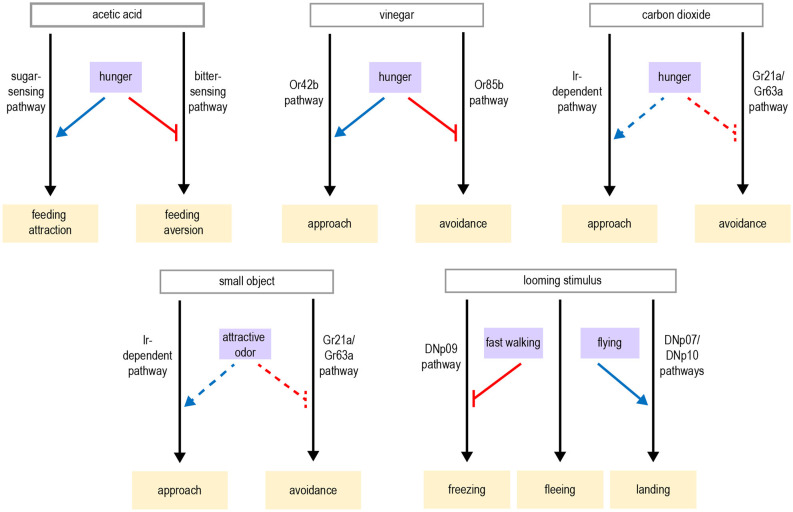
Examples in which the same stimulus can elicit different behavioral responses through competing pathways that are gated by state or context. Black arrows depict neural circuits generating behavior; blue arrows depict excitatory modulation whereas red arrows depict inhibitory modulation. Dashed lines indicate putative types of modulation that have not been experimentally confirmed. For example, it is not known whether fast walking switches the behavioral response to carbon dioxide by enhancing the pathway promoting approach or suppressing the pathway for avoidance.

The examples of behavioral flexibility that we have described primarily fall under three categories: (1) gain changes modulating the strength of a behavior, (2) tuning changes modulating the preferred range of stimuli that produce a response, and (3) a behavioral switch that changes the response entirely (Table 1). Shared principles may underlie these different categories of behavioral flexibility. For example, a behavioral switch can result from gain changes when there are two competing pathways that promote different behaviors (Figure 7). Enhancing or suppressing the output of either pathway beyond a certain threshold can shift their balance and thereby elicit a switch in the behavioral response. This mechanism underlies the hunger-dependent switch in the acetic acid response, the behavioral state-dependent responses to looming stimuli, and many of the learning-dependent changes that rely on MB output.

**Table 1:** Examples of behavioral flexibility described in this review.

Behavior	Modulator	Direction/type of change	Neuronal target of modulation	Modulators involved
**Gain changes**			
Vinegar attraction	Hunger	↑	Or42b- and Or85b-expressing OSNs	Insulin, sNPF, tachykinin
Food-seeking (yeast)	Hunger	↑	MB circuits	Dopamine, NPF, sNPF, serotonin, insulin, allatostatin A
Sugar attraction	Hunger	↑	Sugar-sensing neurons	Dopamine, NPF
Bitter aversion	Hunger	↓	Bitter-sensing neurons	AKH, sNPF, octopamine
Locomotor activity	Hunger	↑	Octopaminergic neurons	AKH, insulin
Aversion to high salt concentrations	Salt deprivation	↓	Downstream of ppk23^glut^ taste neurons	
Yeast consumption	Protein deprivation	↑	DA-WED cells; widespread changes in SEZ	Dopamine
Sugar consumption	Protein deprivation	↓	DA-WED cells	Dopamine
Yeast consumption (by females)	Mating	↑	Putative motor areas of SEZ	Sex peptide, octopamine
Salt consumption (by females)	Mating	↑		Sex peptide
Courtship of female (by males)	Long-term sexual arousal	↑	DANs, P1 neurons	NPF, dopamine
Visual pursuit of female (by males)	Short-term sexual arousal	↑	LC10a neurons	
Sexual receptivity (by females)	Mating	↓	pC1 neurons, vpoDNs	Sex peptide
Egg-laying (by females)	Mating	↑	pC1 neurons, oviDNs	Sex peptide
Sugar consumption (by starved flies)	Water deprivation	↓	ISNs	AKH
Motion sensing*	Walking, flying	↑	HS and VS cells	Octopamine
Feeding initiation	Walking	↓	Mechanosensory interneurons	
Walking	Extended proboscis state	↓		
Carbon dioxide avoidance	Vinegar	↓	DANs, glutamatergic MBONs	Dopamine
Sugar attraction	Yeast odor	↑	Downstream of Or35a-expressing OSNs	
Sugar attraction	Mechanosensation	↑	Downstream of hair plate mechanosensory neurons	
Sugar attraction	Cool temperatures	↓	Downstream of bitter-sensing and mechanosensing neurons	
Learned odor response	Reconsolidation (re-exposure to CS-)	↓ if not properly reconsolidated	Recurrent MB circuits	Dopamine
Learned odor response	Extinction (re-exposure to CS+ alone)	↓	Recurrent MB circuits	Dopamine
Expression of memory for odor-sugar association	Latent inhibition (pre-exposure to CS+)	↓	MB circuit	Dopamine
Expression of memory for odor-sugar association	Hunger	↑	DANs innervating MB	Dopamine, NPF
Learned odor association with sugar (in flies starved after training)	Sleep	↑	MB circuit	Dopamine, NPF
**Tuning changes**			
Temperature preference	Hunger	Shift to lower temperatures	AC cells	
Salt preference	Mating (in females)	Shift to higher concentrations		
Horizontal motion-sensing*	Walking	Shift to higher frequencies	HS cells	
Vertical motion-sensing*	Flying	Tuning broadened toward higher frequencies	VS cells	
**Switch in behavior**			
Acetic acid taste response	Hunger	Switch from aversion to attraction	Downstream of sugar- and bitter-sensing neurons	
Choice between feeding and mating (in males)	Hunger	Choice switches from courtship to feeding	TyrR^PLP^ neurons, P1 neurons	Tyramine
Preference for light	Wing clipping or gluing	Switch from attraction to aversion		Dopamine, Octopamine
Response to looming stimulus	Fast walking	More likely to flee than freeze	Downstream of DNp09 neurons	
Response to looming stimulus	Flying	Switch from escape to landing	Upstream of DNp07 and DNp10	Octopamine
Carbon dioxide	Fast walking, flying	Switch from avoidance to approach		Octopamine
Takeoff response to looming stimulus	Context (looming speed)	Switch from long to short takeoff mode	LC4 and LPLC2 visual neurons, giant fiber neurons	
Steering response toward aversive wind and attractive visual cue	Context (presence of both cues together)	Switch from either aversion/ attraction to turning sequence	
Small visual object	Attractive odor	Switch from avoidance to attraction	Motion-sensitive visual pathway	Octopamine
Odor approach or avoidance	Associative learning	Induce approach or avoidance	KC-MBON synapses in MB	Dopamine
Learned odor response	Reversal learning	Switch response to CS+ vs. CS-	Recurrent MB circuits	Dopamine

*These are not technically examples of behavioral modulation, but modulations of sensory processing that may relate to perception. OSNs, olfactory sensory neurons; sNPF, short neuropeptide F; NPF, neuropeptide F; AKH, adipokinetic hormone; MB, mushroom body; DANs, dopaminergic neurons; MBONs, MB output neurons; AC, anterior cells. Up or down arrows represent an increase or decrease in the behavior, respectively.

Moreover, examples of behavioral flexibility that we currently describe as gain changes may actually represent a behavioral switch if a broader set of behaviors were monitored. For instance, the suppression of PER during walking, or the suppression of courtship after males have recently copulated, likely allows for other behaviors to be expressed instead. It is important for an animal to adequately prioritize mutually exclusive goals. Recent studies investigating the behavioral competition, such as choosing between feeding and mating, highlight the interactions between different behaviors and their underlying circuits. As the field moves toward studying broader repertoires of behavior in more naturalistic paradigms, more of these interactions will likely emerge.

Overall, *Drosophila* has proven to be an excellent model system for dissecting the neural circuits underlying behavioral flexibility. Astounding progress has been made over the last decade in characterizing the synaptic wiring diagram of the fly nervous system and developing increasingly sophisticated genetic tools for circuit analysis. In parallel, methods for behavioral analysis and quantification have drastically improved (Pereira et al., 2020). These tools, combined with our ever-improving estimation of the fly’s behavioral complexity, open the door to obtaining a comprehensive understanding of the molecular and neural circuit mechanisms underlying behavioral flexibility. This knowledge may in turn inform our understanding of the circuit mechanisms underlying behavioral inflexibility that occurs in psychiatric disorders (Barker et al., 2015; Gruner and Pittenger, 2017; Volkow et al., 2017).

## Author Contributions

All authors contributed to the article and approved the submitted version.

## Conflict of Interest

The authors declare that the research was conducted in the absence of any commercial or financial relationships that could be construed as a potential conflict of interest.

## Publisher’s Note

All claims expressed in this article are solely those of the authors and do not necessarily represent those of their affiliated organizations, or those of the publisher, the editors and the reviewers. Any product that may be evaluated in this article, or claim that may be made by its manufacturer, is not guaranteed or endorsed by the publisher.
